# Publication rate in preclinical research: a plea for preregistration

**DOI:** 10.1136/bmjos-2019-100051

**Published:** 2020-08-27

**Authors:** Mira van der Naald, Steven Wenker, Pieter A Doevendans, Kimberley E Wever, Steven A J Chamuleau

**Affiliations:** 1 Cardiology, University Medical Centre, Utrecht, The Netherlands; 2 Regenerative Medicine Center Utrecht, University Medical Centre, Utrecht, The Netherlands; 3 Netherlands Heart Institute, Utrecht, The Netherlands; 4 Systematic Review Centre for Laboratory animal Experimentation, Department for Health Evidence, Radboud Institute for Heath Sciences, Radboudumc, Nijmegen, Gelderland, The Netherlands

**Keywords:** preregistration, preclinicaltrials.eu, translational research, publication rate, publication bias

## Abstract

**Objectives:**

The ultimate goal of biomedical research is the development of new treatment options for patients. Animal models are used if questions cannot be addressed otherwise. Currently, it is widely believed that a large fraction of performed studies are never published, but there are no data that directly address this question.

**Methods:**

We have tracked a selection of animal study protocols approved in the University Medical Center Utrecht in the Netherlands, to assess whether these have led to a publication with a follow-up period of 7 years.

**Results:**

We found that 60% of all animal study protocols led to at least one publication (full text or abstract). A total of 5590 animals were used in these studies, of which 26% was reported in the resulting publications.

**Conclusions:**

The data presented here underline the need for preclinical preregistration, in view of the risk of reporting and publication bias in preclinical research. We plea that all animal study protocols should be prospectively registered on an online, accessible platform to increase transparency and data sharing. To facilitate this, we have developed a platform dedicated to animal study protocol registration: www.preclinicaltrials.eu.

**Strengths and limitations of this study**

- This study directly traces animal study protocols to potential publications and is the first study to assess the number of animals used and the number of animals published.
- We had full access to all documents submitted to the animal experiment committee of the University Medical Center Utrecht from the selected protocols.
- There is a sufficient follow-up period for researchers to publish their animal study.
- Due to privacy reasons, we are not able to publish the exact search terms used.
- A delay has occurred between the start of this project and time of publishing, this is related to the political sensitivity of this subject.

## Introduction

Biomedical research is performed to gain an understanding of (patho)physiological mechanisms and ultimately to use this knowledge to develop new therapies for patients. However, limitations in the design and reporting of experiments are known to cause avoidable research waste,^1 2^ and it has been estimated that 85% of all research costs and efforts is wasted.^3–5^ One important factor leading to avoidable waste is publication bias, in which the outcome of a study influences the chance of publishing. This has been recognised as an important problem in the biomedical sciences for several decades.^6 7^ The systematic over-representation of statistically significant study results leads to an overestimation of effect sizes, threatens the validity of systematic reviews and meta-analyses and can influence the development of guidelines and recommendations, or the decision to proceed to a clinical trial.^8 9^

The publication rate (ie, the percentage of conducted studies that is eventually published) is an important indicator of publication bias, and has been extensively studied for clinical trials, for example, by tracking studies from their initiation to publication or non-publication. Such studies report a wide variation in publication rates, ranging from 12.5%^9^ to 93%,^10^ depending on for example the source of identification of the trials (eg, institutional ethics committee approvals vs entry in a clinical trial protocol registry), the trial phase and the source of funding.^9–12^ The statistical significance of the trial outcomes is associated with both the publication rate and the time to publication.^11 13^

Although not as extensively evaluated, there is also reason for concern regarding the selective publication of preclinical animal studies.^14–16^ As in clinical research, systematic review and meta-analysis has been instrumental in making publication bias in animal research transparent. Between 46% and 62% of preclinical systematic reviews find evidence of publication bias.^16^ In preclinical neurology research, the number of animal studies reporting statistically significant beneficial treatment effects far exceeds the expected number of animals studies with such positive results.^17^ Furthermore, an estimated 14% of animal studies in stroke is performed but not published, possibly causing a relative overestimation of the overall effect of treatment of 31%.^18^ In a survey among Dutch animal researchers, respondents estimated the publication rate of animal studies to be on average 50% in non-for-profit organisations, and 10% in for-profit organisations.^1^ Important reasons of non-publication indicated by the respondents were lack of statistical significance, the opinions of supervisors and peer reviewers, and technical problems during the experiment.

However, compared with clinical studies, measuring publication and reporting bias (selective reporting of results) in animal studies directly by assessing their publication rate from initiation is more difficult, because few accessible registries of animal study protocols exist and publications of animal studies rarely refer to a study protocol. Here, we present the first study investigating the publication rate in animal research, by tracking a set of research protocols from their approval by an animal ethics committee, to publication or non-publication.

## Methods

### Study protocol selection

We tracked animal studies performed at three research departments at the University Medical Center Utrecht, for which study applications were approved by the animal ethics committee in 2008 or 2009. Applications from commercial parties were not included. At that time all applications for animal studies in the Netherlands were approved by local institutional animal experiment committees. Applications are confidential, and mainly consist of the study protocol, which includes background information, hypotheses, a sample size calculation and a detailed description of the experimental procedures. We were granted access to applications only after consent from at least one of the researchers listed on the application.

### Searching and selecting publications

**Figure 1:**
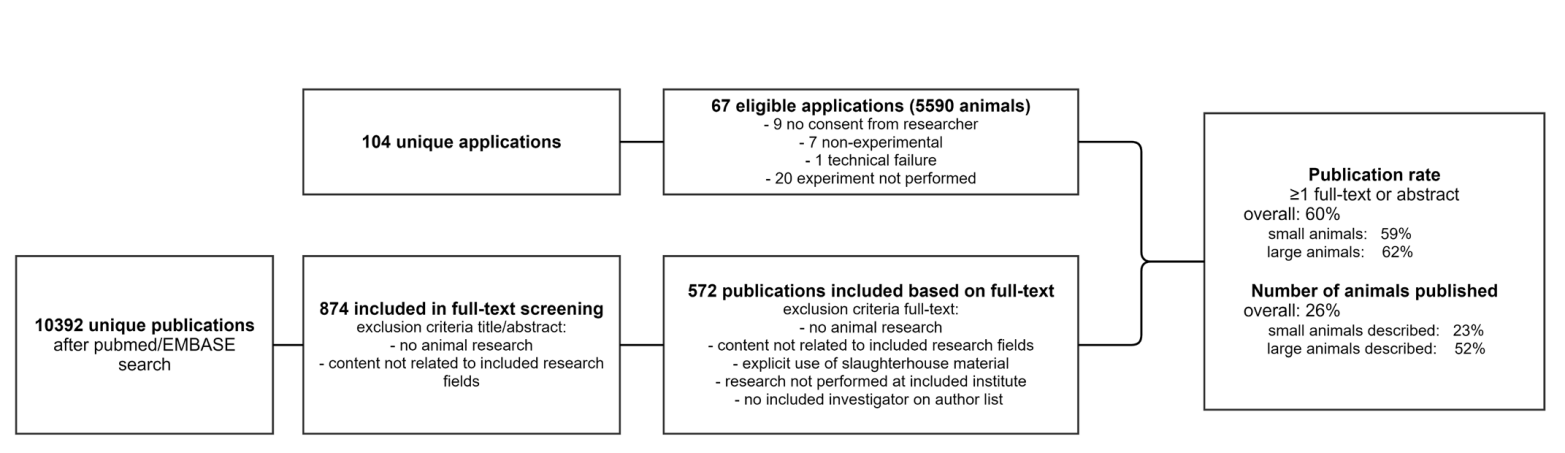
Flow chart showing the total number of applications, the number of included applications, the number of publications in the PubMed/EMBASE search, in the full-text screening and those included in the linking to the applications.

We performed systematic searches using the names of all investigators listed on the 67 applications, in PubMed and EMBASE on 14 March 2016. The search string therefore included all researchers’ names. For example, for PubMed the search was as follows: “last name researcher #1 initials”[author] OR “last name researcher #2 initials”[author] OR “last name researcher #3 initials”[author] OR” etc. Search results were limited to articles published after 01/01/2007. The search results were screened for eligibility based on their title and abstract by either MvdN or SW. Based on a title/abstract screening, publications on animal studies related to any of the three involved research departments were included. Publications were included if they were (1) primary reports on animal study data and (2) related to any of the three involved research departments. Full-text screening was performed in duplicate by two independent reviewers (MvdN and SW). Exclusion criteria were as follows: (1) not a primary report of an animal study, (2) not related to (experimental) cardiology or medical physiology, (3) use of slaughterhouse material only, (4) the animal experiment committee reported in the article was not from the institute of interest, (5) study was performed in accordance to non-Dutch legislation, (6) the animal ethics committee application number format did not match that of the institute of interest and (7) the author’s full name did not match the name of the researcher on the application (eg, same initials, but different full first name). Included articles were sorted per animal species (figure 1).

### Matching of study protocol to publications

Two reviewers (MvdN and SW) independently identified whether the application has led to a publication. For each application, possible publications were identified in the publication database by matching animal species, involved researchers with the author list and performing a detailed comparison of the research question, animal model, intervention(s) and experimental procedures described in the application versus the publication. Discrepancies were solved by a third reviewer (KW).

### Endpoints

The aim of this study was to determine the publication rate of animal studies within the three participating departments. Publication rate is assessed using the following definitions: (1) the number of applications which led to ≥1 publications (full-text only), (2) the number of applications which led to ≥1 publications (abstracts included) and (3) the number of animals published as an percentage of the total number of animals sacrificed for the performed studies.

### Count of animals

Data from the local animal welfare bodies were used to identify the number of animals sacrificed per application. The total of number of animals (including those reported as ‘excluded’ or ‘deceased’) reported in each matched publication was extracted by two independent researchers (MvdN and SW). When publications lacked details on the exact number of animals used for a specific experiment, we used the biggest number mentioned (eg, when a group size was mentioned as a range from 3 to 5, we noted 5 animals).

### Limited updated search

On 7 June 2019, the search was repeated in PubMed and Embase. For PubMed, results were filtered by publication date after 1 March 2016 and species ‘Other Animals’. For Embase, the following filters were applied: publication year 2016–2019 and study types: ‘animal experiment’, ‘animal model’, ‘animal tissue’, ‘disease model’, ‘feasibility study’, ‘in vivo study’, ‘model’, ‘mouse model’, ‘nonhuman’ and ‘preclinical study’. Title and abstract screening and matching to research applications were performed as described above by one researcher (SW). This resulted in 1286 unique publications. After title and abstract screening 133 publications remained. The publications were only compared with research applications for which no publications had been identified yet. Ultimately, no new matches were made.

### Post hoc survey

A post hoc survey was conducted in March 2018 among the involved researchers. Goal of this survey was to verify if the tracing was done correctly, if any publications were missing and to assess why data were not published. Researchers were sent an overview of the applications, including the number of animals used according to the institutional data as well as the publications identified by our search. They were asked (1) if the found publications were correct, (2) if all data were published, (3) if not, why data were not published, (4) if the study was explorative or confirmative and (5) if the study result was significant or not-significant.

## Results

A total of 104 unique applications were approved by the three selected research departments in 2008 and 2009 at our institution. Part of the protocols that were approved in 2008 or 2009 were continuations of research that was originally started in 2007. These applications were included. We obtained consent to access the study protocols from at least one of the researchers listed on the applications for 95 (91%) of these applications. Seven applications were excluded based on their non-experimental character (ie, applications for training or educational purposes), and one application was not accessible due to a technical failure. Local animal welfare bodies documented the number of animals sacrificed per application. According to this data, 20 of the 87 (23%) remaining applications were never carried out. Thus, study protocols from 67 applications were included in our analysis (figure 1). There were four applications for which assessment by a third reviewer (KW) was needed to determine whether the publication matched the application. Assessment by a third reviewer (KW) was also needed three times to decide on the number of animals mentioned in a publication.

A total of 30 full-text papers and 41 conference abstracts were found to be produced from these 67 applications. Our search identified at least one full-text publication resulting from the research application for 46% (31/67) of the applications. Sixty per cent (40/67) was published when conference abstracts were also taken into account. After stratifying for species, the publication rate (full text or abstract) for small animal models (mice, rats and rabbits) was 59% (24/41), compared with 62% (16/26) for large animal models (pigs, dogs and sheep; figure 1).

According to institutional administration, a total of 5590 animals were used in the 67 applications. In total, 26% (1471/5590) of the animals were described in the publications resulting from these applications. This percentage was considerably lower for small animals (23% (1190/5014) of animals published), than for large animals (52% (299/576) of animals published; figure 1).

The 40 applications that were published accounted for 79% of the total animals used (4402/5590). Out of these published applications, reports on small animals described on average 30% of the animals used in the applications (1190/3979, range 6%–100%). For studies involving large animals, this was on average 71% (299/423 range 8%–100%).

The average time between approval of a project by the animal ethics committee and the first resulting publication was 30.7 months (median 27.5). In this sample, the longest time between approval of a project and the first publication (either full text of abstract) was 65 months. In one case, the first full-text manuscript was published after 90 months, but an abstract had already been published after 35 months.

A post hoc survey conducted in March 2018 among the involved researchers. The survey was sent out to all researchers that gave permission for their 67 included applications. We received a response for 53 (79%) of the applications. We discovered one publication that was not identified by our search; this publication is included in our analysis. One survey participant informed us that a manuscript was in preparation, but had not yet been published; this manuscript is not included in our analysis. The most frequently reported reasons for non-publication were a lack of statistical significance, the study being a pilot study and technical problems with the animal model.

## Conclusion and discussion

With this study we attempt to determine the publication rate of animal research. To the best of our knowledge, this is the first report tracking the number of animals used, providing a percentage of animal published. The results show that 60% of the animal studies were ultimately published, but a considerable number of the animals used is not reported in these publications, as only 26% of the used animals were reported.

### Sample size

Although our sample size was relatively small, we believe that these data are likely to be representative of the field of preclinical research (and not specific to our institution). These findings are consistent with a previously published survey among 454 laboratory animal researchers in the Netherlands, that estimated that approximately 50% of animal experiments is published.^1^ A recent study reporting the publication rate of animal studies in Germany showed a publication rate of 67%, which is in line with our findings.^19^ The most frequently reasons not to publish mentioned in our post hoc survey are similar to the reasons reported in a previous survey (lack of statistical significance, technical problems and objections from supervisors and peer reviewers).^1^ Although we expected the publication rate of animal studies to be lower the numbers, we found are comparable to publication rates reported in the clinical domain.

### Animal tracking

Our post hoc survey identified only a single publication not identified by our search, indicating that our search and matching approach was able to correctly identify and match the vast majority of publications to the corresponding application. We noticed that publications often included experimental groups that could not have originated from the same application. These animals were not included in the number of published animals. Because results from separate research applications (with similar animal experiments) are frequently combined in a single report we cannot exclude that some of the animals described in the identified publications originated from other applications (which, eg, could have been performed before our study period). In that case the publication rate may be lower than reported here.

### Follow-up period

**Figure 2:**
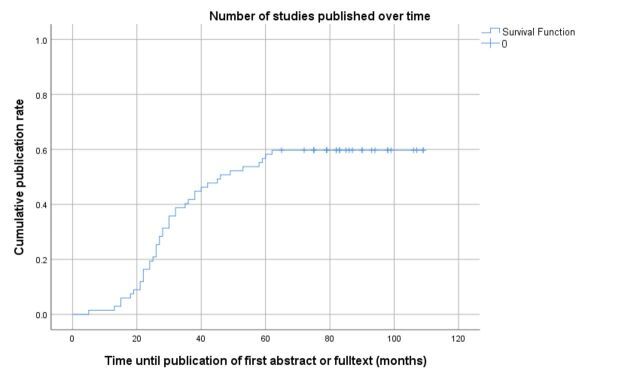
Kaplan-Meier curve with time between approval by the animal ethics committee until first publication (abstract or full text).

The research applications analysed here were performed in 2007, 2008 and 2009, which allowed researchers up to 7 years to publish their findings. It is therefore unlikely that results from these experiments will be reported on in future publications if they have not been shared with the research community thus far. Concordantly, the survival publication curve (figure 2) reaches a plateau after 60 months. Due to the ethically and thus politically sensitive nature of our findings, over 3 years have passed since the systematic search and submission of the manuscript. However, as argued above, it is unlikely that the publication rate has significantly increased in the intervening period. This is also supported by the fact that our post hoc survey (performed in 2018) did not identify any papers published after our study period and the fact that our additional search did not identify any new matches.

Over the past decade, many studies have highlighted the importance of good research practices, among which is the sharing of data and publishing negative results.^20–26^ Although there is growing awareness of the importance of good research practices, we are not aware of any data indicating that publication rates have significantly increased between 2008 and present day. Especially in the preclinical domain, there is a paucity of data available on this subject which this report in part is meant to address.

### Sharing of data

We believe that results of virtually all animal experiments should be shared with the research community. Animal studies are performed for the benefit of human health, and the ethical justification for the use of animals rests on those benefits. Increasing transparency and data sharing in animal research are essential to ensure a valuable contribution of animal experiments to advancing human health(care). The sharing of non-significant results or technical failures is important for scientific progress, for example, by improving methodology of animal models, as well as to prevent research waste in the form of unnecessary replications by others who are unaware of your results. There may also be a (perceived) lack of interest from scientific journals to publish non-significant data (lack of statistical significance was named as an important reason not to publish).

### Preregistration

Prospective registration of animal study protocols—as is already common practice in the clinical arena—may increase sharing of data. If all animal studies are preregistered, researchers can use the animal study protocol database for a comprehensive overview of all experiments that have been performed to aid in answering research questions and designing new studies. It may allow researchers to identify colleagues who are working on the same topic or with experience with similar animal models and it can provide a platform where researchers can share unpublished data. Furthermore, prospective registration can improve study design by emphasising the importance of rigour. Finally, it creates transparency around key elements of the experiment that was originally planned (eg, sample size calculations primary outcome) and enables comparison of the original protocol with the study as it was ultimately reported.^27–29^

### Implementation of preclinicaltrials.eu

To facilitate preregistration, we developed www.preclinicaltrials.eu: the first online accessible, international register dedicated to the (pre)registration of animal studies (launched 11 April 2018).^28 30 31^ The register aims to provide a comprehensive listing of animal studies to help avoid unplanned duplication, minimise publication bias and increase transparency. The platform allows registrants to link their protocols to published or unpublished data, thus enabling others to identify unpublished studies and data, for example, for the purpose of a systematic review or meta-analysis.

All stakeholders involved in animal studies and translational research (ie, researchers, institutions, funders and journals) should underscore the importance of preregistration of animal studies in order to incorporate this in routine practice. In this respect, it is very promising that the Dutch parliament recently unanimously accepted a motion declaring that all animal studies should be (prospectively) registered, and that all their results should be made publicly available. In addition, multiple policy makers, Dutch institutes (including the Netherlands Heart Institute) and funding agencies are taking steps towards implementation of preregistration. Utrecht University and University Medical Centre Utrecht have decided to make such preregistration mandatory. Various international scientific communities, such as the Transnational AllianCe for regenerative Therapies In Cardiovascular Syndromes (TACTICS) consortium and several working groups of the European Society for Cardiology, are committed to implement preregistration within their research fields, journal editors are discussing the possibilities to implement preregistration within their author guidelines and other countries and researchers are discussing and working on animal study registration.^28 31 32^ In the meantime, we encourage individual researchers to take responsibility and actively contribute to prospective registration of preclinical trials.
